# Draft genome sequence of the peptaibol producer *Trichoderma asperellum* TRC-03

**DOI:** 10.1128/mra.00133-26

**Published:** 2026-04-20

**Authors:** Pamela Alfaro-Vargas, Daniela Wicki-Emmenegger, Esteve Mesén-Porras, Cristina Vargas-Chacón, Oscar Bravo Bonilla, Priscila Chaverri, Max Chavarría

**Affiliations:** 1 Centro Nacional de Innovaciones Biotecnológicas (CENIBiot), CeNAT-CONARE 541212, San José, Costa Rica; 2 Instituto Nacional de Innovación y Transferencia en Tecnología Agropecuaria (INTA), San José, Costa Rica; 3 Department of Natural Sciences, Bowie State University 1887, Bowie, Maryland, USA; 4 Centro de Investigaciones en Productos Naturales (CIPRONA), Universidad de Costa Rica 27915, San José, Costa Rica; 5 Escuela de Química, Universidad de Costa Rica 27915, San José, Costa Rica; Rochester Institute of Technology, Rochester, New York, USA

**Keywords:** *Trichoderma asperellum*, peptaibols, short-chain-length peptides, antifungal

## Abstract

We present the genome sequence of *Trichoderma asperellum* TRC-03, an isolate obtained from an agricultural soil and whose capacity to produce peptaibols was previously demonstrated. The *Trichoderma* genome showed a size of 36,213,208 bp and a total of 55 metabolic genes related to peptaibol biosynthesis.

## ANNOUNCEMENT

Several fungal species, including those of the genus *Trichoderma*, have demonstrated the ability to produce peptaibols, short-chain-length peptides of 5–20 residues, most non-proteinogenic (1–3). The interest in these biomolecules lies in their different biological activities, including antifungal (4, 5), anticancer (6), anti-*Toxoplasma* (7), insecticidal (8), and antiplasmodial (9). Here, we report the genome of *Trichoderma asperellum* TRC-03, a strain whose peptaibol-rich extracts showed the ability to inhibit pathogens such as *Alternaria alternata*, *Botrytis cinerea*, *Colletotrichum gloeosporioides*, and *Fusarium oxysporum* that affect important crops (4). Strain TRC-03 was isolated from agricultural soil from Desamparados, San José, Costa Rica, using the procedure described by Cai and Druzhinina (10).

**Fig 1:**
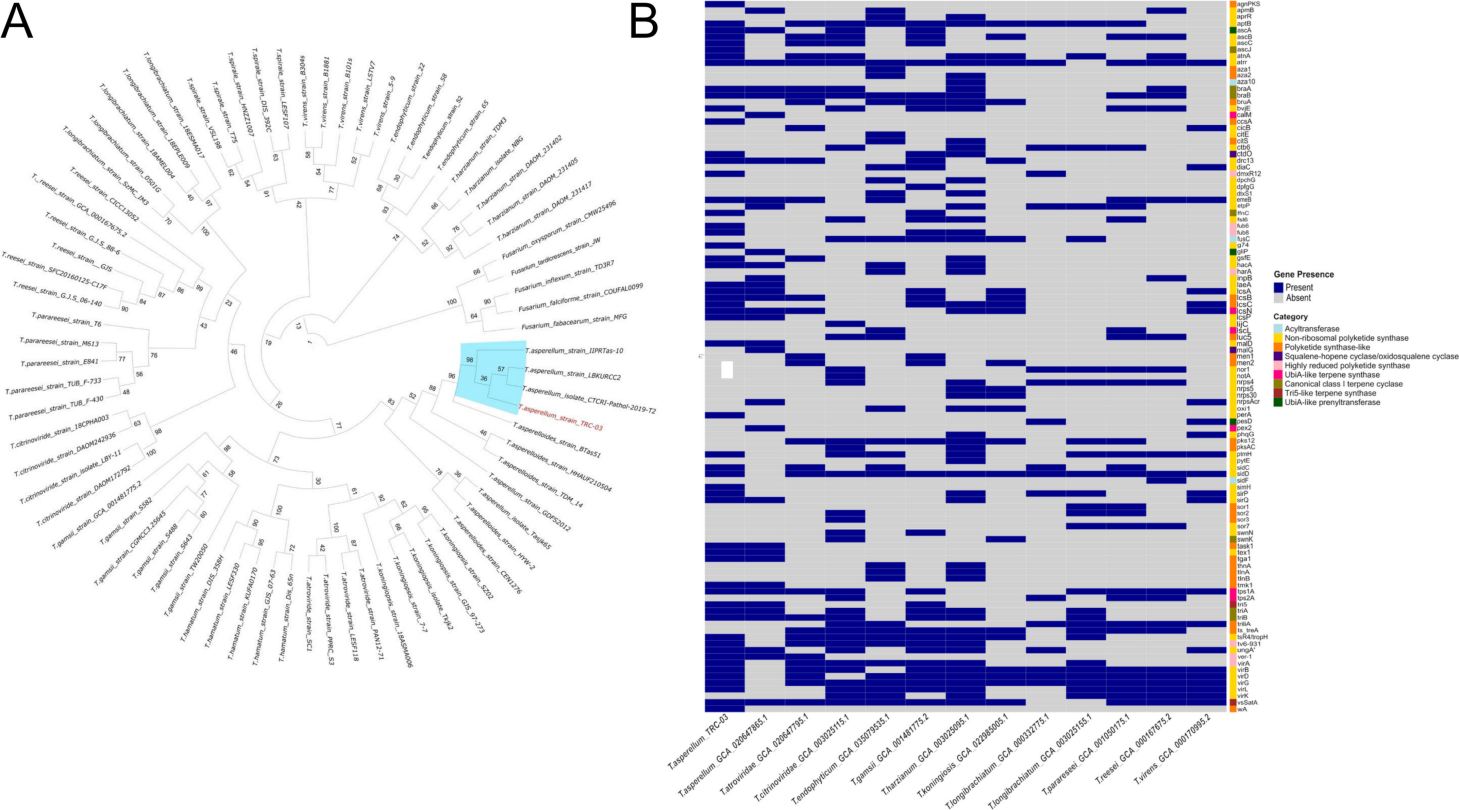
Phylogenetic tree and genes involved in peptaibol biosynthesis in *T. asperellum* TRC-03. (**A**) A fungal phylogenetic tree was constructed using the TEF-1α gene, with the TRC-03 strain located within the *T. asperellum* clade (light blue shaded). The phylogenetic tree with ITS sequences (not shown) also groups strain TRC-03 within the *T. asperellum* clade. (**B**) Genes related to peptaibol biosynthesis identified in *T. asperellum* TRC-03 and other *Trichoderma* species. The color scale in the heatmap indicates the absence (gray) or presence (dark blue) of 144 distinct genes associated with peptaibol biosynthesis. The gene symbols (right *Y*-axis) are categorized into nine biosynthetic gene clusters (BGCs), each represented by a different color (legend on the right). Gene symbols and descriptions of the protein products were obtained from the manually curated fungal BGC database and the fungal genome mining tool, part of the FunBGCeX module for predicting novel BGCs. The following biosynthetic genes are part of or code for non-ribosomal peptide synthase (NRPS): *apmB* (NRPS *L*-2-aminopimelic acid B), *aprR* (short-chain dehydrogenase asperipin-2a biosynthesis cluster protein R), *aptB* (NRPS adenine phosphoribosyltransferase B), *ascB* (NRPS-like carboxylic acid reductase ascofuranone/ascochlorin B), *ascC* (NRPS-like carboxylic acid reductase ascofuranone/ascochlorin C), *atnA* (NRPS aspercryptin A1), *atrr* (NRPS-like glycine betaine reductase transcription factor ATRR), *bvjE* (NRPS-like brevijanazine E), *citE* (NRPS citrate synthase subunit E), *cicB* (NRPS-like carboxylic acid reductase cichorine B), *ctb6* (short-chain dehydrogenase cercosporin toxin biosynthesis 6), *drc13* (short-chain dehydrogenase DNA replication checkpoint protein 13), *diaC* (short-chain dehydrogenase dibenzodioxocinone C), *dpfgG* (NRPS DDP fungal gene cluster G), *dtxS1* (NRPS destruxin synthase 1), *emeB* (NRPS emericellamide B), *etpP* (NRPS epipolythiodioxopiperazine protein P), *fsl6* (NRPS-like carboxylic acid reductase fusarielin 6), *g74* (NRPS protein kinase G74), *gsfE* (short-chain dehydrogenase griseofulvin E), *hacA* (PKS harzianic acid A), *laeA* (uncharacterized NRPS putative methyltransferase), *lcsA* (NRPS leucinostatin A), *lcsP* (NRPS leucinostatin P), *lijC* (short-chain dehydrogenase lijiquinone C), *malD* (short-chain dehydrogenase malic enzyme D), *nor1* (short-chain dehydrogenase norsolorinic acid ketoreductase 1), *notA* (short-chain dehydrogenase notoamide A), *nrps4* (NRPS 4), *nrps5* (NRPS 5), *nrps30* (NRPS 30), *nrpsAcr* (NRPS acremopeptaibol), *oxi1* (short-chain dehydrogenase oxidative signal-inducible 1), *perA* (NRPS peramine synthesis), *phqG* (short-chain dehydrogenase paraherquamide G), *ptmH* (short-chain dehydrogenase platensimycin H), *pytE* (short-chain dehydrogenase pyranterreone E), *sidD* (NRPS siderophore D), *simH* (NRPS simvastatin H), *sirP* (NRPS sirodesmin P), *sirQ* (short-chain dehydrogenase sirodesmin Q), *sor7* (short-chain dehydrogenase sorbicillinol 7), *tex1* (short-chain dehydrogenase TRE X complex subunit 1), *tsR4*/*tropH* (NRPS aldehyde dehydrogenase temperature-sensitive ribosomal protein four and gene cluster for tropolone H), *ungA*′ (NRPS unguisin A′), *virB* (short-chain dehydrogenase virulence factor B), *virD* (short-chain dehydrogenase virulence factor D), *virG* (short-chain dehydrogenase virulence factor G), *virK* (short-chain dehydrogenase virulence factor K), and *virL* (short-chain dehydrogenase virulence factor L). The following genes are part of or code for canonical class I terpene synthases: *ascJ* (short-chain dehydrogenase ascJ), *braA* (brasilane glycoside A synthase), *braB* (brasilane B glycosyltransferase), *ffnC* (terpene synthase fosfonochlorin C), *swnK* (swainsonine K synthase), *triA* (trichothecene A synthase), and *triB* (trichothecene B synthase). *atrR* codes for NRPS-like glycine betaine reductase ATRR. The following genes code for acyltransferases: *aza10* (acyltransferase azasperpyranone 10), *fusC* (acyltransferase fusidic acid C), and *sidF* (acyltransferase siderophore F). The following genes code for polyketide synthase-like enzymes (PKS-like): *agnPKS* (agnestin PKS), *aza1* (PKS azasperpyranone 1), *aza2* (non-reducing PKS azasperpyranone 2), *bruA* (PKS brunneoxanthone A), *ccsA* (PKS copper chaperone for superoxide dismutase A), *citS* (PKS citrate synthase subunit S), *lcsB* (PKS leucinostatin B), *lcsC* (PKS leucinostatin C), *luc5* (PKS lucilactaene 5), *men1* (short-chain dehydrogenase menisporopsin A), *men2* (PKS menisporopsin A), *pks12* (PKS 12), *pksAC* (PKS ascochitine), *sor1* (PKS sorbicillinol 1), *sor2* (PKS sorbicillinol 2), *sor3* (PKS sorbicillinol 3), *task1* (PKS derived from *Trichoderma asperellum* specific mitogen-activated protein kinase 1), *tga1* (PKS derived from *Trichoderma viride* F-534 G alpha subunit gene), *thnA* (PKS tetrahydroxynaphthalene reductase A), *tlnA* (PKS tricholignan A), *tlnB* (PKS tricholignan B), *tmk1* (PKS derived from *Trichoderma* sp. mitogen-activated protein kinase 1), *triliA* (PKS *Trichoderma* sp. biosynthetic cluster tenellin synthase A), ts_*treA* (PKS derived from *Trichoderma* sp. periplasmic trehalase A), and *wA* (PKS for wall pigment secretion). The following genes are part of or code for HR PKS: *cmtA* (HR PKS copper metallothionein A), *dmxR12* (short-chain dehydrogenase dimeric xanthone biosynthesis cluster protein R12), *fub6* (HR PKS *Fusarium* sp. fusaric acid 6), *fub8* (HR PKS carboxylic acid reductase acid 8), *harA* (HR PKS harzianopyridone A), *scyPKS* (HR PKS scytalidin polyketide synthase), *tv6-931* (HR PKS from *Trichoderma virens* PKS cAT domain 6-931), *ver-1* (short-chain dehydrogenase versicolorin A), and *virA* (HR PKS virulence factor A). The following three genes code for squalene-hopene cyclase/oxidosqualene cyclase: *ctdO* (short-chain dehydrogenase cycloaddition to form the bicyclo[2.2.2]diazaoctane O), *malG* (squalene-hopene cyclase transport of maltose G). These four genes code for ubiA-like prenyltransferase: *ascA* (Ubi-like prenyltransferase ascofuranone/ascochlorin A), *gliP* (ubiA-like gliotoxin biosynthesis protein P), *pesD* (UbiA-like prenyltransferase peptide synthase D). The following genes code for UbiA-like terpene synthases: *calM* (UbiA-like prenyltransferase calmodulin M), *lcsL* (UbiA-like terpene synthase leucinostatin L), *lcsN* (UbiA-like terpene synthase leucinostatin N), *pex2* (*Penicillium expansum* short-chain dehydrogenase peroxisomal membrane protein 2), *tps1A* (UbiA-like terpene prenyltransferase synthase 1A), and *tps2A* (UbiA-like terpene prenyltransferase synthase 2A). These two genes code for tri5-like terpene synthases: *tri5* (Tri5-like terpene synthase), *vsSatA*: (tri5-like terpene *Verticillium sakurae* streptothricin acetyltransferase A).

The isolate stored in glycerol 15% vol/vol at −80°C was grown in potato dextrose agar (7 days, 25°C). DNA extraction was performed using cetyltrimethylammonium bromide protocol (11) and sequenced through Oxford Nanopore (ONT) and Illumina NovaSeq technologies. For ONT sequencing, the library was prepared using the SQK-LSK109 Ligation Kit (ONT, UK). A total of 12 µL of the library was loaded onto a MinION flow cell and sequenced with a MinION MK1B device (ONT) for 72 hours, following the standard protocol (12). Raw data were base called with Dorado v0.8.1 (13) to convert POD5 to FASTQ. For Illumina sequencing, a paired-end library was prepared and loaded onto the NovaSeq 6000 (PE150). Raw data quality was assessed with FastQC v0.11.9 (14). Low-quality reads were filtered based on a Phred score of 20 for ONT and 28 for Illumina data. Adapter sequences were filtered using Trimmomatic v0.36C (15). A *de novo* hybrid assembly was performed with MaSuRCA v7.0.7 (16), and its quality was assessed with QUAST v5.2.1 (17). Genome annotations were obtained with BRAKER v3.06 (18) and compared to the *T. asperellum* reference CGF_020647865 using Companion v2.2.11 (19). Default parameters were used for all software. Genome assembly statistics and annotation features are presented in Table 1. A phylogenetic tree for translation elongation factor 1α (TEF-1α) was constructed with Sanger sequences using Phylosuite v1.2.3 (20). Sequences were aligned with MAFFT (21) and trimmed with TimAI, and a concatenated tree was generated with IQ-TREE 2 v1.6.12 (22). Results (Fig. 1A) confirm that the TRC-03 strain is closely related to *T. asperellum* species. The biosynthetic gene clusters of TRC-03 and other peptaibol producers were analyzed with FunBGCeX v0.0.2 (23). A data frame was then constructed to identify genes involved in peptaibol biosynthesis. A matrix for plotting a heatmap was built using dplyr v1.1.4 (24), reshape v1.4.4 (25), tidyverse 2.0.0 (26), and pheatmap v1.0.12 (27). Figure 1B presents a heatmap of genes involved in peptaibol synthesis across *Trichoderma* species. In the TRC-03 genome, 55 metabolic genes were predicted.

**TABLE 1:** Annotation and genome assembly statistics of the peptaibol producer *T. asperellum* TRC-03 isolated from a Costa Rican agricultural soil

Isolate feature	*Trichoderma asperellum* TRC-03
BioProject accession no.	PRJNA1172749
Assembly accession no.	JBLGTG000000000
No. of raw reads (Illumina)	1,403,177,100
No. of paired-end reads (Illumina)	4,677,257
No. of raw reads (ONT)	1,734,967,438
Genome size (bp)	36,213,208
No. of contigs	381
No. of predicted genes	10,529
Coverage (1×) (bp)	47.90
G + C content (%)	48.79
N_50_ (bp)	186,091
L_50_ (bp)	48
No. of tRNAs	171
No. of rRNAs	57
No. of ncRNAs	3
No. of snRNAs	12

## Data Availability

The whole-genome sequencing of the TRC-03 strain was submitted to NCBI/GenBank under accession no. JBLGTG000000000, Bioproject no. PRJNA1172749, and BioSample no. SAMN44845004. The raw reads were deposited with SRA numbers SRX26384498 (ONT) and SRX26384497 (Illumina). The current version referred to in this paper is identified as version 1.0.
